# Electrochemical Reduction of CO_2_ With Good Efficiency on a Nanostructured Cu-Al Catalyst

**DOI:** 10.3389/fchem.2022.931767

**Published:** 2022-07-07

**Authors:** Juqin Zeng, Micaela Castellino, Marco Fontana, Adriano Sacco, Nicolò B. D. Monti, Angelica Chiodoni, Candido F. Pirri

**Affiliations:** ^1^ Center for Sustainable Future Technologies @POLITO, Istituto Italiano di Tecnologia, Turin, Italy; ^2^ Department of Applied Science and Technology, Politecnico di Torino, Turin, Italy

**Keywords:** carbon dioxide utilization, electrochemistry, aluminum, copper, synthetic gas, formic acid, electrolyte

## Abstract

Carbon monoxide (CO) and formic acid (HCOOH) are suggested to be the most convenient products from electrochemical reduction of CO_2_ according to techno-economic analysis. To date, tremendous advances have been achieved in the development of catalysts and processes, which make this research topic even more interesting to both academic and industrial sectors. In this work, we report nanostructured Cu-Al materials that are able to convert CO_2_ to CO and HCOOH with good efficiency. The catalysts are synthesized *via* a green microwave-assisted solvothermal route, and are composed of Cu_2_O crystals modified by Al. In KHCO_3_ electrolyte, these catalysts can selectively convert CO_2_ to HCOOH and syngas with H_2_/CO ratios between 1 and 2 approaching one unit faradaic efficiency in a wide potential range. Good current densities of 67 and 130 mA cm^−2^ are obtained at −1.0 V and −1.3 V vs. reversible hydrogen electrode (RHE), respectively. When switching the electrolyte to KOH, a significant selectivity up to 20% is observed for C_2_H_4_ formation, and the current densities achieve 146 and 222 mA cm^−2^ at −1.0 V and −1.3 V vs. RHE, respectively. Hence, the choice of electrolyte is critically important as that of catalyst in order to obtain targeted products at industrially relevant current densities.

## Introduction

Electrochemical conversion of CO_2_ into valuable chemicals and fuels has attracted tremendous interest, since it possesses advantageous properties such as the utilization of green electrolytes, the high tunability of products, the potential implementation of electricity from renewable energy sources and the mild reaction conditions (Gurudayal et al., 2017; Huan et al., 2019; Sacco et al., 2020). Despite significant prospect, the electrochemical CO_2_ reduction reaction (CO_2_RR) encounters many challenges associated to high energy barriers, multiple parallel reactions and competitive hydrogen evolution reaction (HER) (Yin et al., 2019). Consequently, a wide range of chemicals such as carbon monoxide (CO), formate (HCOO^−^), methane (CH_4_), methanol (CH_3_OH), ethane (C_2_H_6_), ethylene (C_2_H_4_) and ethanol (C_2_H_5_OH) is identified as the CO_2_RR products, and the formation of H_2_ is almost inevitable in aqueous electrolytes (Bagger et al., 2017; Zhao et al., 2018; Ren et al., 2019; Zeng et al., 2021a; Zeng et al., 2021b; Jeng et al., 2022; Zhou et al., 2022). Among these species, CO and its mixture with H_2_ (H_2_/CO syngas) have high relevance for the chemical industry (Nielsen et al., 2018; Zeng et al., 2020a). Particularly, several processes were already well-established for the generation of fuels and fine-chemicals from syngas (Hernández et al., 2017). Formate (HCOOH and HCOO^−^) is also an important chemical raw material for various industries such as leather, rubber, medicine, fiber. Moreover, it can be used as fuel in a direct formic acid fuel cell and as an excellent carrier of stored H_2_ (Yu and Pickup, 2008; Eppinger and Huang, 2017). Most particularly, a techno-economic analysis suggests that the short-chain simple building-block molecules CO and HCOOH are currently the most compelling CO_2_RR products (Bushuyev et al., 2018).

The acquisition of targeted products calls for suitable catalysts able to selectively drive the CO_2_RR, and great efforts have been dedicated to the study of electrocatalysts in recent years (Nitopi et al., 2019; Li et al., 2022). Among the monometals, gold (Au), silver (Ag) and zinc (Zn) are selective for CO formation, while lead (Pb), mercury (Hg), indium (In), bismuth (Bi) and tin (Sn) are remarkably selective for HCOOH production (Bagger et al., 2017). Many bimetallic catalysts also have good potential to catalyze the CO_2_RR toward desired products, since the electronic and geometric structures of these materials are highly tailorable. As widely reported, Cu-Zn (Moreno-García et al., 2018; Zeng et al., 2020b), Cu-Sb (Li et al., 2020; Zeng et al., 2022a) and Cu-Sn (Zeng et al., 2018; Yoo et al., 2020) are demonstrated to selectively produce CO, while most of Sn and Bi bimetallic materials (Hou et al., 2019; Wu et al., 2021) show good HCOOH selectivity. The most intensively studied bimetallic material system is Cu-Sn that can efficiently produce CO and HCOOH at controllable proportions by engineering the Cu/Sn ratio of the catalyst surface (Rabiee et al., 2020). Cu-Ag materials are another class of bimetallic catalysts that are widely reported for CO_2_RR. Herzog et al. showed a catalyst with 5 at% Ag on Cu_2_O nanocubes, achieving a two-fold increase in the Faradaic efficiency for C_2+_ liquid products (30% at −1.0 V_RHE_) (Herzog et al., 2021). Y.C. Li et al. developed a bimetallic Ag/Cu catalyst that achieved a good Faradaic efficiency of 41% toward ethanol at 250 mA cm^−2^ and −0.67 V_RHE_, leading to a cathodic side (half-cell) energy efficiency of 24.7% (Li et al., 2019). On contrast, Cu-Al materials are scarcely studied for the electrochemical CO_2_RR. Honma et al. (Iwase et al., 2022) studied two-dimensional Cu- and Al-based layered double hydroxides with different Cu/Al ratios and sheet sizes, and obtained the best CO faradaic efficiency (FE) of 42% and the highest formate selectivity of 22% at 50 mA cm^−2^ under galvanostatic conditions. Sargent et al. (Zhong et al., 2020) identified a Cu-Al catalyst using density functional theory calculations in combination with active machine learning, which efficiently reduced CO_2_ to C_2_H_4_ with a high FE over 80%. Very recently, A.S. Rasouli et al. synthesized porous Ga-doped CuAl catalysts able to disrupt carbon-carbon coupling and shift the selectivity from C_2_H_4_ to CH_4_ while maintaining low hydrogen evolution activity (Sedighian Rasouli et al., 2022).

Based on the literature, Cu-Al materials show tremendous potential for CO_2_RR application, while the reported performance are inconsistent, particularly in the selectivity. Inspired by these reported works, we continue to explore Cu-Al catalysts for CO_2_RR. Various Cu-Al materials with different Cu/Al ratios were synthesized *via* one-step microwave-assisted method using copper acetate and aluminum nitrate as metal precursors and ethylene glycol as solvent. The obtained Cu-Al catalysts show highly nanostructured surface that are rich of active sites, contributing to the CO_2_RR at high reaction rates. An optimal Cu/Al ratio results in good selectivity for the CO_2_RR, with a FE of 47% for HCOOH and 24.5% for CO in KHCO_3_ electrolyte. When considering syngas as target instead of CO, the current efficiency is almost 100%, with only a small amount of C_2_H_4_. When changing the electrolyte from KHCO_3_ to KOH, enhanced C_2_H_4_ selectivity as well as electrode activity are observed.

## Experimental

### Materials

Copper acetate (Cu(CH_3_COO)_2_, 99.9%), aluminum nitrate (Al(NO_3_)_3_·9H_2_O, 98%), potassium bicarbonate (KHCO_3_, 99.7%), ethylene glycol (EG, 99.8%), Nafion® 117 solution (5 wt%) and isopropanol were purchased from Sigma-Aldrich. Unless otherwise specified, all the materials were used as received.

### Synthesis of the Catalysts

The Cu-Al catalysts were fabricated through a modified microwave-assisted solvothermal route (Zeng et al., 2020a). Typically, 0.9 g of Cu(CH_3_COO)_2_ and a certain amount of Al(NO_3_)_3_·9H_2_O were dissolved in 40 ml of EG and 5 ml of H_2_O. Different amounts of Al(NO_3_)_3_·9H_2_O were used in order to tune the Cu/Al ratios of the materials, as shown in Table 1. After 10 min of vigorous agitation, the mixture was transferred into a Teflon vessel (volume 100 ml). The Teflon vessel was put in a microwave oven (Milestone STARTSynth, Milestone Inc., Shelton, Connecticut) and connected to pressure and temperature probes. The mixture was irradiated for 2 min at 900 W (T_Max._ = 220°C) and then was cooled to ambient temperature. The precipitate was separated by centrifuge and washed twice with H_2_O and once with ethanol. The powder sample was finally obtained by vacuum drying at 60°C overnight. The Cu-Al samples were denoted as Cu_2_O and Cu_2_O-Al-x, where x equals to 1, 2, 3, 5 and 9, respectively.

**TABLE 1:** Preparation of materials with various ratios of Cu and Al precursors.

Sample	Cu(CH_3_COO)_2_	Al(NO_3_)_3_·9H_2_O
	(mg)	(mg)
Cu_2_O	900	0
Cu_2_O-Al-1	900	180
Cu_2_O-Al-2	900	370
Cu_2_O-Al-3	900	560
Cu_2_O-Al-5	900	930
Cu_2_O-Al-9	900	1670

### Physical and Chemical Characterizations of the Catalysts

Field emission scanning electron microscopy (FESEM, ZEISS Auriga) was used to evaluate the morphology of the catalysts. X-ray diffraction (XRD) was performed on the powder samples by using a PANalytical X’Pert Pro instrument (Cu-Kα radiation, 40 kV and 30 mA) equipped with an X’Celerator detector. The Rietveld refinement of XRD patterns was carried out with MAUD software (Ferrari and Lutterotti, 1994). Line broadening due to crystallite size distribution and micro-strain was modeled with the “Distribution” function implemented in MAUD, coupled with either isotropic (samples Cu_2_O-Al-1, Cu_2_O-Al-2, Cu_2_O-Al-5) or anisotropic (samples Cu_2_O, Cu_2_O-Al-3, Cu_2_O-Al-9) size-strain model developed by N. C. Popa (Popa, 1998). The contribution to line broadening by the X-ray diffractometer was determined by refining the XRD pattern of the LaB6 NIST standard sample, measured in identical conditions.

X-ray photoelectron Spectroscopy (XPS) has been performed by means of a PHI Versaprobe 5000 spectrometer (Physical Electronics, Chanhassen, MN, USA), equipped with a monochromatic Al K-alpha X-ray source (1486.6 eV), to check the composite surface chemical composition. A circular spot of 100 μm in diameter was selected to gather the photoelectron signal for both the high resolution (HR) and the survey spectra. All samples were subjected to a combined electron and Ar ion gun neutralizer system, to decrease the electrical charging effect during the analysis. The semi-quantitative atomic concentration and fitting procedures were acquired using CasaXPS 2.3.23 dedicated software (Casa Software Ltd.,Wilmslow, UK). All core-level peak energies were referenced to C1s peak at 284.5 eV and the background contribution in HR scans was subtracted by means of a Shirley function. In order to compare our results with a proper reference sample, we have also prepared a Cu metal foil by sputtering it inside the XPS apparatus with Argon ions at 2 kV for 5 minutes, to remove any native oxide from the top surface.

### Preparation of the Electrodes

The preparation process plays an important role in maximizing the behaviors of the electrodes (Martinez Crespiera et al., 2016; Vankova et al., 2017). In this work, the electrodes were prepared by drop-casting the as-prepared catalyst onto a carbon paper. In a typical preparation, 10 mg of catalyst, 1.0 mg of carbon black (CB, Shawinigan Black AB50) and 90 μl of Nafion^®^ 117 solution were well mixed with 150 μl of isopropanol. The mixture was sonicated for 30 min until a uniform slurry was obtained. The slurry was then coated onto a carbon paper (GDL, SIGRACET 28BC, SGL Technologies). The obtained electrode was dried at room temperature overnight to evaporate the solvents.

### Electrochemical Tests and Product Analysis

Electrochemical impedance spectroscopy (EIS) measurements were performed in a three-electrode single-compartment cell at room temperature with a Metrohm Autolab electrochemical workstation. The working electrode was an as-prepared electrode with a geometric area of about 0.2 cm^2^. A Pt wire was used as the counter electrode and Ag/AgCl (3 M Cl^−^) as the reference. The electrolyte was a CO_2_-saturated 0.5 M KHCO_3_ aqueous solution (pH 7.8). EIS measurements were performed at various potentials of −0.6, −0.8, −1.0, and −1.2 V vs. reversible hydrogen electrode (RHE) with an AC signal of 10 mV of amplitude and 10^–2^–10^4^ Hz frequency range. Unless otherwise specified, all potentials refer to RHE and are shifted according to Nernst equation: E (V vs. RHE) = E (V vs. Ag/AgCl) + E°_Ag/AgCl_ + 0.059 * pH, where E (V vs. RHE) is the reported potential value, E (V vs. Ag/AgCl) is the potential value vs. reference electrode, E°_Ag/AgCl_ is the standard potential of reference electrode and pH is the pH value of the electrolyte.

CO_2_ electrolysis was carried out by applying chronoamperometric (CA) technique with a CHI760D electrochemical workstation. The comparison of various electrodes was carried out in 0.5 M KHCO_3_ electrolyte, while the optimal electrode was further studied in 2.0 M KHCO_3_ (pH 8.4) and 1.0 M KOH electrolyte (pH 12) in a customized three-compartment flow cell, as shown in Scheme S1 and thoroughly described in our previous work (Zeng et al., 2022a). A Ag/AgCl (1 mm, leak-free LF-1) was used as the reference electrode and inserted in the catholyte. A Pt foil (Goodfellow, 99.95%) was used as the counter electrode and immersed in the anolyte. The working electrode was a catalyst-coated carbon paper with a geometric area of 1.5 cm^2^. In the case of KHCO_3_ electrolyte, a proton exchange membrane (Nafion™ Membrane N117, Sigma-Aldrich) was used to separate the anodic and cathodic compartments. Both catholyte and anolyte were circuited at 2 ml min^−1^ during the test. A constant CO_2_ flow of 10 ml min^−1^ was purged through the anolyte in order to maintain a constant pH. A CO_2_ flow of 25 ml min^−1^ was maintained at the gas compartment of the cathodic side in order to deliver CO_2_ as reactant and bring out products. When 1.0 M KOH electrolyte was used, an anion exchange membrane (Sustainion^®^ 37–50, Dioxide materials) was employed to separate the anodic and cathodic compartments. Both catholyte and anolyte passed through the corresponding compartments at 2 ml min^−1^. A CO_2_ flow of 25 ml min^−1^ was maintained at the gas compartment of the cathodic side in order to supply CO_2_ reactant and bring out products.

The potential was corrected by compensating the ohmic potential drop, of which 85% by the instrument (iR-compensation) and 15% by manual calculation. Gas-phase products were analyzed on-line by a micro gas chromatograph (µGC, Fusion^®^, INFICON) with two channels containing a 10 m Rt-Molsieve 5A column and an 8 m Rt-Q-Bond column, respectively. Both channels were equipped with a micro thermal conductivity detector. Liquid products were analyzed by a high-performance liquid chromatograph (Shimadzu HPLC) with a UV-Vis Detector set at 210 nm by using a ReproGel (300 mm × 8 mm) column, with 9.0 mM H_2_SO_4_ (flow rate of 1.0 ml min^−1^) as mobile phase.

The FE for each product was calculated by dividing the charge needed to produce the actual determined amount of this product by the total charge consumed during a corresponding reduction period, as shown in Eq. 1:

$$\mathrm{FE}=\frac{nNF}{Q}$$ (1)

where N is the amount of a detected product (number of moles, mol); *n* is the number of electrons required to obtain 1 molecule of this product (*n* = 2 for CO, HCOOH and H_2_ formation, *n* = 12 for C_2_H_4_); *F* is the Faraday constant (96485 C mol^−1^); Q is the total charge passed through the system recorded during electrolysis (coulombs, C).

The production rate of a product (mmol h^−1^ cm^−2^) was calculated through Eq. 2:

$$Production\;rate=\frac{j_{total}*FE*t}{nF}$$ (2)

where FE is the faradaic efficiency for the specific product, $j_{\mathrm{total}}$ is the total geometric current density of the electrode, t is a constant of 3600 and F is the Faraday constant (96485 C mol^−1^).

## Results and Discussion

### Physical/Chemical Properties of Cu_2_O-Al-X Samples

**FIGURE 1:**
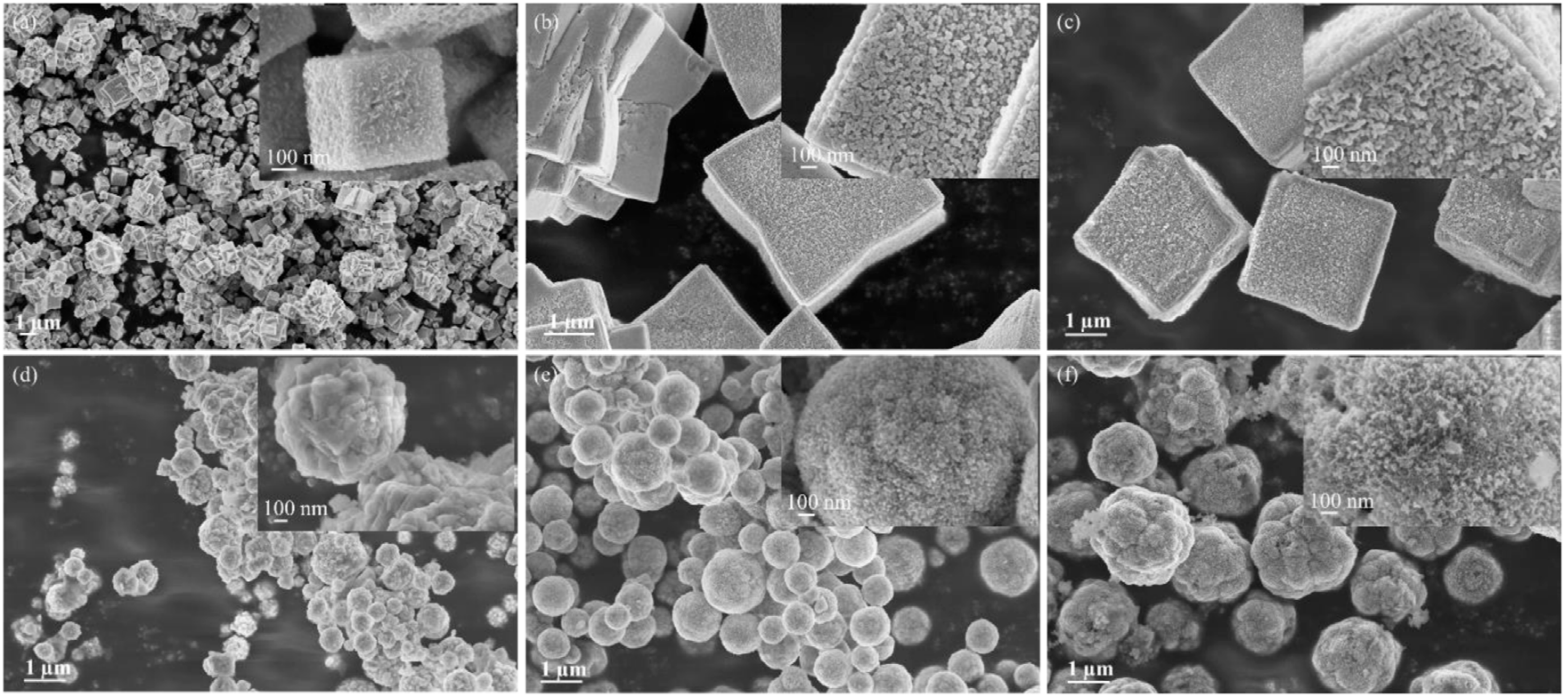
FESEM images of Cu_2_O-Al-x samples. **(A)** Cu_2_O; **(B)** Cu_2_O-Al-1; **(C)** Cu_2_O-Al-2; **(D)** Cu_2_O-Al-3; **(E)** Cu_2_O-Al-5 and **(F)** Cu_2_O-Al-9.

Figure 1 shows the morphology of the as-prepared samples. The Cu_2_O sample consists of submicrometric cubic and micrometric polyhedral aggregates within a size range of 0.3–0.7 µm with irregular external surface populated by nanoplatelets (Figure 1A and inset of Figure 1A). With the addition of lower amounts of Al (samples Cu_2_O-Al-1 and Cu_2_O-Al-2), the size of the particles increases to 2.5–3.5 µm, while the cubic morphology with nanostructured external surface is retained (Figures 1B,C). The addition of high amounts of Al shows a remarkable effect on the morphology of samples Cu_2_O-Al-3, Cu_2_O-Al-5 and Cu_2_O-Al-9: micrometric spheres with variable size distribution (Figures 1D–F) are formed and each sphere consists of loosely packed nano-sized particles (inset of Figures 1D–F). The aggregates increases in size as raising the amount of Al precursor for sample Cu_2_O-Al-3, Cu_2_O-Al-5 and Cu_2_O-Al-9. The Cu_2_O-Al-3 shows an aggregate size of 0.3–0.7 µm, and it increases to 0.7–1.6 µm for Cu_2_O-Al-5 and 1.6–2.3 µm for Cu_2_O-Al-9 sample. It is worth to note that the effect of Al addition on the morphology is similar with that of the doping with Sn (Deng et al., 2015) and Sb (Zeng et al., 2022a). The reason of this phenomenon is not clearly stated in the literature and remains unclear in this study. Further work is needed in order to clarify this point.

EDX analysis was performed to roughly evaluate the chemical composition of the materials. As listed in Table 2, C, O, Cu and Al are the main detected elements. The C signal is produced by the carbon tape used for FESEM sample preparation and by the adventitious carbon contamination. The atomic ratio of Cu and Al decreases as lowering that of their salts in the precursor solution from Cu_2_O-Al-1 to Cu_2_O-Al-3, while it does not decrease for Cu_2_O-Al-5 and Cu_2_O-Al-9 when further increasing the Al salt in the precursor solution. However, ICP analysis shows a gradual increase in the weight percentage of Al in the samples with increasing the Al salt in the precursor solution. This outcome indicates the inhomogeneity of the materials, that is, a minor phase rich of Al could exist. This hypothesis is confirmed by detailed FESEM and EDX analysis. As shown in Figure S1, scarce and large agglomerations are present in the Cu_2_O-Al-5 and Cu_2_O-Al-9 samples, and they are mainly composed of Al and O with low Cu percentages.

**TABLE 2:** Element distribution by EDX on various Cu_2_O-Al-x samples.

Sample	C	O	Al	Cu	Cu/Al	Al^a^
	(at.%)	(at.%)	(at.%)	(at.%)	(at./at.)	(wt.%)
Cu_2_O-Al-1	33.6	26.8	0.7	38.9	56	0.4
Cu_2_O-Al-2	38.6	25.9	0.9	34.6	39	0.9
Cu_2_O-Al-3	21.1	29.5	2.8	46.6	16	2.5
Cu_2_O-Al-5	17.0	35.7	2.8	44.4	16	3.8
Cu_2_O-Al-9	49.7	26.7	1.2	22.4	19	7.1

a The weight percentage of Al is quantified by ICP.

**FIGURE 2:**
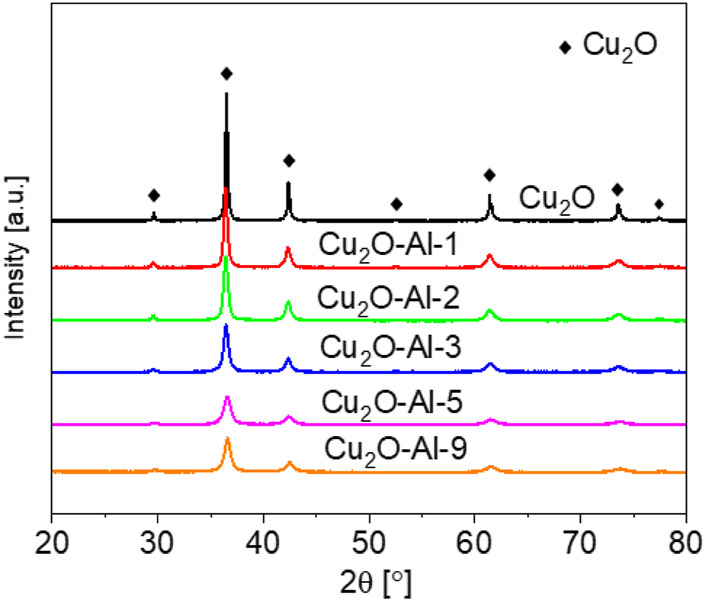
XRD patterns of Cu-Al samples.

To identify the crystalline phase compositions of the materials, XRD analysis has been employed on the powder samples. As shown in Figure 2, all peaks are associated to the (110), (111), (200), (211), (220), (311) and (222) planes of Cu_2_O with a cubic structure (Crystallography Open Database ID: 9007497, cubic unit cell, lattice constant a = 4.2685 Å, P n -3 m space group) for all samples. It is interesting to notice that no Al-containing crystalline phase is identified, suggesting that Al could be successfully incorporated in the Cu_2_O crystalline structure. Moreover, there is no significant contribution from amorphous phases, since the XRD patterns do not show the typical large bumps of amorphous materials (Bates et al., 2006).

Further information on the microstructure is obtained by the Rietveld method, which allows the estimation of crystallite size and micro-strain contributions to the XRD pattern. The refined parameters are provided in Table 3, while the Rietveld refinement plots are shown in the Supporting Information (Supplementary Figure S2). Concerning the lattice constant of the cubic unit cell, it does not show significant deviation with respect to the reference value of a = 4.2685 Å, since for any sample the variation is comparable to 0.1% or lower and there is no correlation with the Al content. The estimated crystallite size, however, shows clear correlation with the Al content: a higher amount of Al precursor ratio results in a smaller average crystallite size. This effect is particularly noticeable for samples Cu_2_O-Al-5 and Cu_2_O-Al-9, which exhibit average crystallite sizes lower than 50 nm. Regarding the micro-strain, moderate values in the 10^–4^–10^–3^ range are estimated, which are expected for defective nanostructured materials (Handoko et al., 2016; Andrade et al., 2017). No correlation is found between Al-precursor content and micro-strain values.

**TABLE 3:** Refined parameters of microstructure: lattice constant α, crystallite size and micro-strain.

Sample	α (Å)	Size (nm)	Micro-strain (*10^–4^)
Cu_2_O	4.2660	92–146	2.0–2.4
Cu_2_O-Al-1	4.2677	132	1.0
Cu_2_O-Al-2	4.2686	91	19
Cu_2_O-Al-3	4.2630	84–132	19–24
Cu_2_O-Al-5	4.2699	41	32
Cu_2_O-Al-9	4.2693	12–37	9.8–10

**FIGURE 3:**
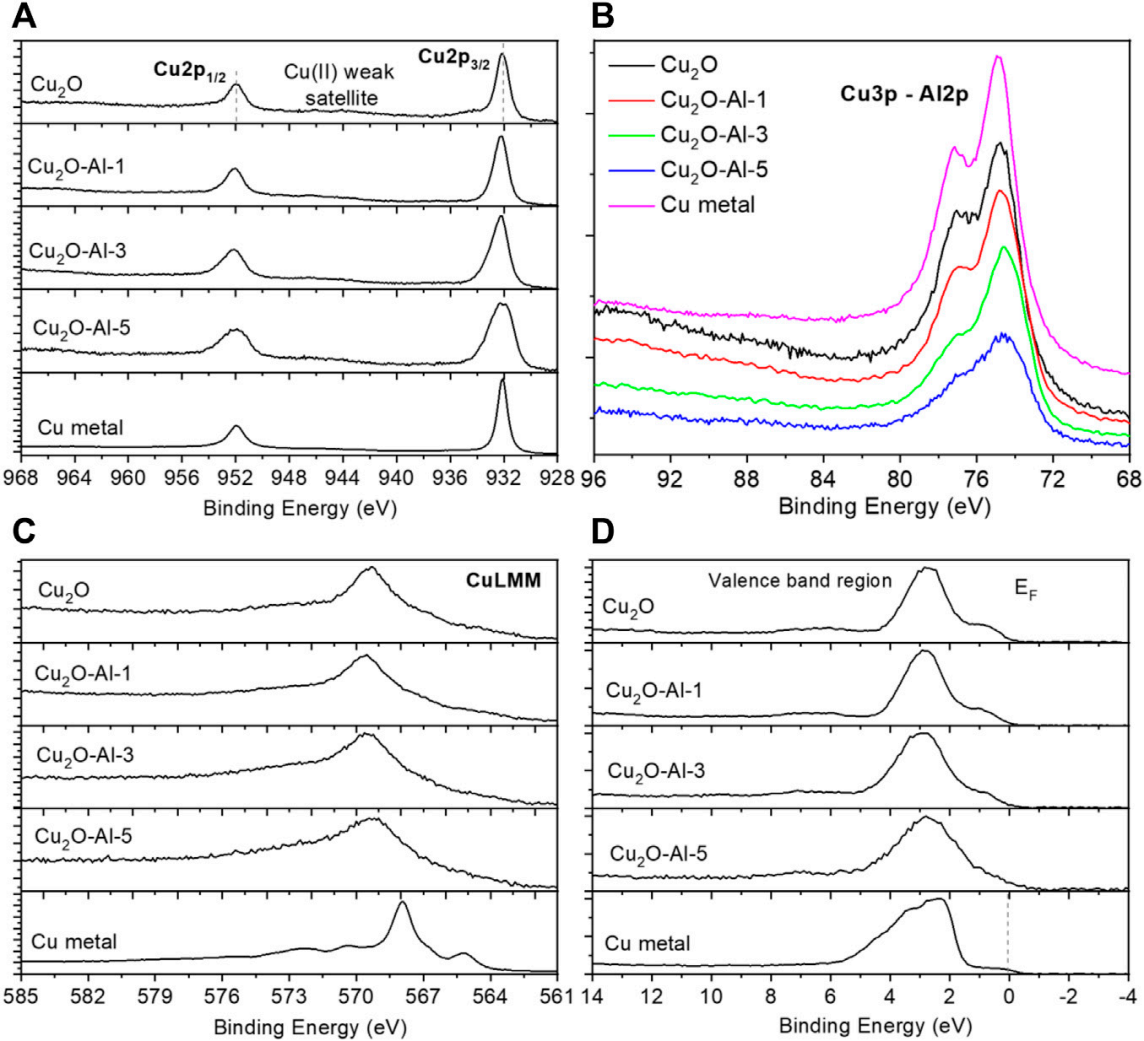
XPS spectra of **(A)** Cu2p doublet, **(B)** Cu3p and overlapping Al2p region, **(C)** CuLMM Auger peaks and **(D)** valence band regions (E_F_ stands for Fermi Energy level) for Cu_2_O, Cu_2_O-Al-1/3/5 and Cu metal reference samples.

XPS analysis has been conducted on selected Cu_2_O-Al-x samples (Cu_2_O-Al-1, Cu_2_O-Al-3 and Cu_2_O-Al-5) and bare Cu_2_O powder. A Cu metallic foil has also been analyzed, in order to obtain reference spectra to be compared with those of the homemade materials. Cu XPS signals have always represented a crucial and quite tricky set of data to be deconvoluted properly, since different Cu oxidation states are not simply recognizable and distinguishable, especially Cu(I) and Cu(0). When Cu(II) is present, a well-defined satellite region appears in the range (940–950) eV, so the Cu(II) is clearly detectable. When a mix of oxidation states exists, an attenuation of the Cu(II) satellite is clearly evident, according to the relative percentage of each species. To have much more information, necessary to obtain a complete scheme of the Cu chemical shifts, we must acquire not only the Cu2p doublet region (see Figure 3A), but also the CuLMM Auger peak (Figure 3C) and the valence band (VB) region as well (Figure 3D). The Auger peak will add new information which can be coupled with the Cu2p doublet position in order to calculate the modified Auger Parameter (Biesinger, 2017), which allows us to distinguish between Cu(0) and Cu(I), among other species and compounds. This latter distinction can be further confirmed by analyzing the shape of the VB region, which extends almost from 0 to 14 eV (Fernandez et al., 2020). Since Al2p signal is completely covered by Cu3p doublet (see Figure 3B), also this latter region has been acquired to get information about Cu and Al at the same time (Al2s signal is completely covered instead by Cu3s peak, region not reported). If we start by looking at the Cu2p doublet region (Figure 3A), we can see that while Cu_2_O sample shows a typical spectrum mainly due to Cu(I) with a small amount of Cu(II), which can be inferred by the small satellite peak at 540–545 eV, Cu_2_O-Al-x samples show a lower satellite, which means that a lower percentage of Cu(II) is expected. To be more precise, we can calculate the ratio between Cu(II) and (Cu(I)+Cu(0)) thanks to the equations presented by M. Biesinger (Biesinger, 2017). The results obtained are reported in Table 4. There is a trend starting from the bare Cu_2_O sample, which possesses the highest amount of Cu(II) = 17%, while samples with Al, at different %, show the same amount of Cu(II) = 8%. Just to compare, also the metal sample has been included, showing a Cu(II) = 0%. If we look instead at the Full Width at Half Maximum (FWHM) parameter for Cu2p_3/2_ peak (see Table 4), we can observe an enlargement of the peak starting from 1.22 eV for bare Cu_2_O till 1.70 eV for Cu_2_O-Al-5. Since the FWHM increase is not related to the appearance of a new component due to a new oxidation state, as verified with the ratio calculated just above, this means that the inclusion of Al atoms in the Cu-based matrix leads to a more “disordered” material, which is reflected in the related peak broadening. Something similar happens in the Cu3p region (see Figure 3B) where we can clearly see not only a broadening of the Cu3p doublet according to the Al content increase, but also the loss of the doublet split (which is visible in the Cu metal reference curve) due to the overlapping between the Cu3p and the underneath Al2p doublet in the region of 72–76 eV.

**TABLE 4:** XPS parameters related to Cu2p3/2 peak FWHM, Cu(II)/Cu(I)+Cu(0) ratio, Modified Auger Parameters calculated for this work and reference values from the literature (Biesinger, 2017) and correlated average oxidation state for Cu, for samples Cu_2_O, Cu_2_O-Al-1/3/5 and Cu metal reference.

Sample	Cu2p_3/2_ FWHM (eV)	Cu(II)/Cu(I)+Cu(0) (%)	Modified auger parameter (eV) [this work]	Modified auger parameter (eV) (Biesinger, 2017)	Average oxidation state (Biesinger, 2017)
Cu_2_O	1.22	17/83	1849.5	1849.2	Cu(I)
Cu_2_O-Al-1	1.27	8/92	1849.3	1849.2	Cu(I)
Cu_2_O-Al-3	1.48	8/92	1849.2	1849.2	Cu(I)
Cu_2_O-Al-5	1.70	8/92	1849.5	1849.2	Cu(I)
Cu metal ref	0.97	0/100	1851.5	1851.2	Cu(0)

We can add another tile to this puzzle by looking at the CuLMM Auger region (Figure 3C), in which we can appreciate the fact that the peak maximum remains constant in its position in all the Cu(I) containing samples (Cu_2_O and Cu_2_O-Al-x), while it changes dramatically when we deal with the Cu metal reference. By calculating and comparing the Modified Auger Parameters for our samples with reference in the literature (Biesinger, 2017), as reported in Table 4, we have a further confirmation that the average oxidation state for Cu_2_O and Cu_2_O-Al-x samples is always Cu(I), while the parameter changes, as expected, for the reference metal sample. A last and final check can be done by looking at the VB region, by comparing firstly the Cu_2_O and the Cu metal curves, as reported by V. Fernandez et al. (Fernandez et al., 2020), since the shape of this region is completely different for this two oxidation state (see Figure 3D). The addition of Al atoms creates a change in the VB region which causes the loss of the well-defined step in 0–2 eV region due to the overlap of Al contribution, which possesses a valence band curve that extends from 0 to 10 eV (Snijders et al., 2002). Concisely, all investigated Cu_2_O-Al-x samples show similar chemical compositions except the different Al percentages on the surface.

### Electrochemical Measurements and Product Analysis

**FIGURE 4:**
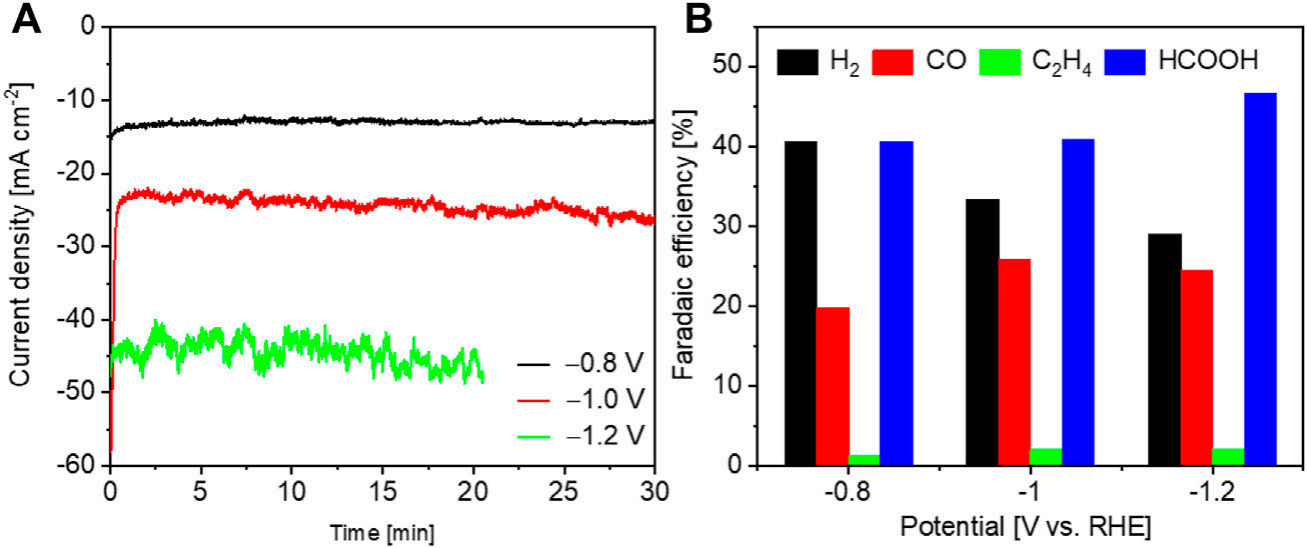
CO_2_ electrolysis on Cu_2_O-Al-3 at various potentials in 0.5 M KHCO_3_. **(A)** CA curves and **(B)** faradaic efficiencies.

CO_2_ electrolysis was firstly compared on various Cu_2_O-Al-x materials in 0.5 M KHCO_3_ electrolyte. Figure 4A reports, as an example, the CA curves at different potentials for Cu_2_O-Al-3 material. Similar curves were obtained for all other electrodes. The current density increases with raising the overpotential, and its oscillation becomes more significant as lowering the potential due to the formation of more gas products. From Figure 4B, it is noticed that CO and HCOOH are the main CO_2_RR products together with a small amount of C_2_H_4_ (FE < 2.1%). The ratio of H_2_/CO varies from 1.1 to 2.0 at the investigated potentials, indicating that this syngas is potentially utilizable for the methanol synthesis (Hernández et al., 2017).

**FIGURE 5:**
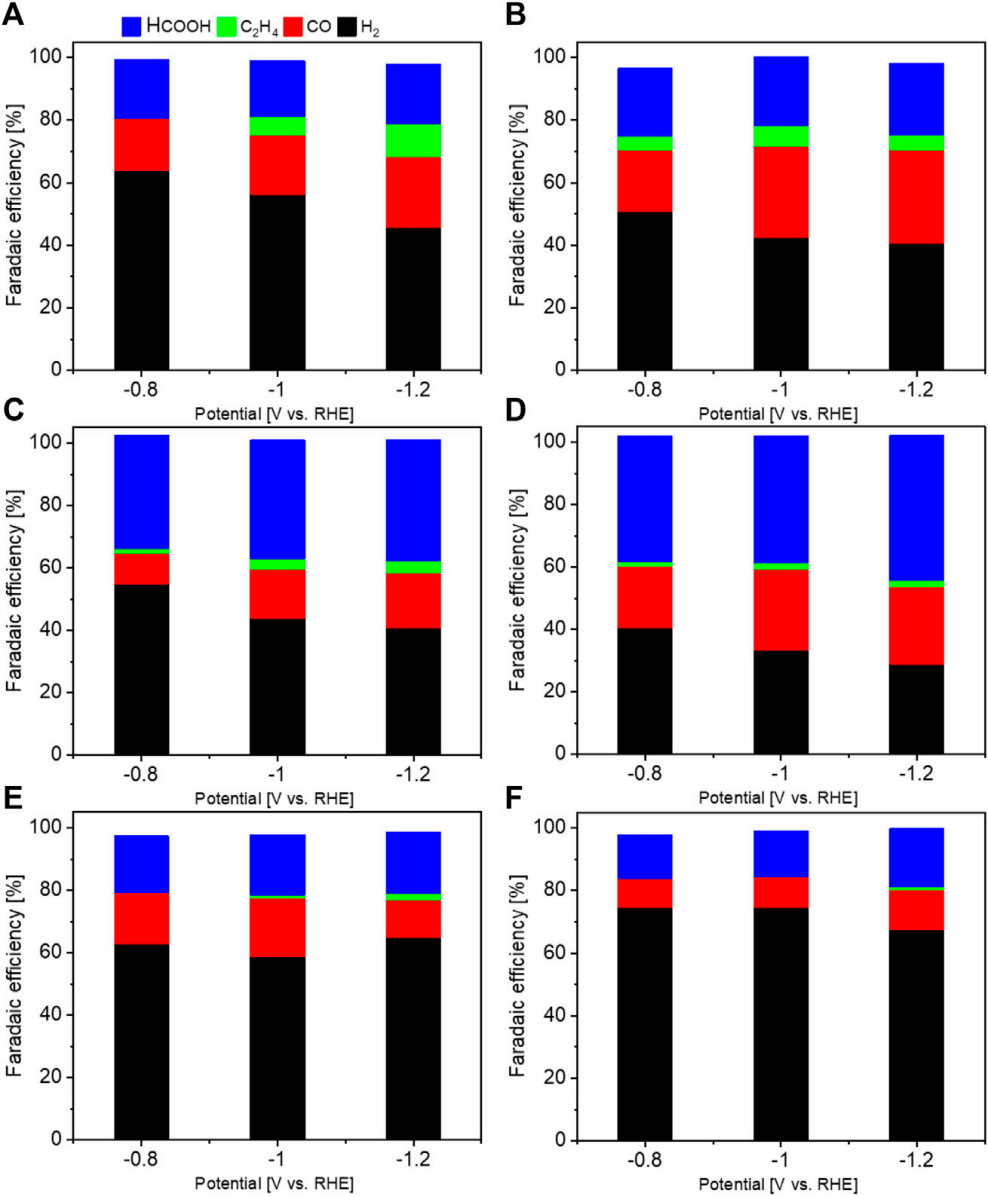
Comparison of the selectivity of different electrodes at various potentials in 0.5 M KHCO_3_: **(A)** Cu_2_O; **(B)** Cu_2_O-Al-1; **(C)** Cu_2_O-Al-2; **(D)** Cu_2_O-Al-3; **(E)** Cu_2_O-Al-5 and **(F)** Cu_2_O-Al-9.

Figure 5 compares the selectivity for main products at different electrodes at various potentials in 0.5 M KHCO_3_. As shown in Figure 5A, Cu_2_O has moderate selectivity for the CO_2_RR, and mainly produces CO (FE 16–23%) and HCOOH (FE ∼19%) with a lower amount of C_2_H_4_ (FE 5–10%). The modest addition of Al slightly promotes the CO_2_RR against the HER on the Cu_2_O electrode (in Figures 5B–D), while further introduction of Al leads to a gradual decrease of CO_2_RR selectivity (Figures 5E,F). The selectivity for C_2_H_4_ production is suppressed on all Cu_2_O-Al samples with respect to that on Cu_2_O electrode, remaining below 2.1%. This outcome is in good agreement with the results reported by Honma et al. (Iwase et al., 2022), while it is inconsistent with those observed by Sargent et al. (Zhong et al., 2020). Honma et al. synthesized two-dimensional Cu- and Al-based layered double hydroxides (Cu−Al/LDHs) using a simple co-precipitation method employing sodium carbonate solutions with different pH and synthesis temperatures. The elemental ratio of Cu and Al was between 1 and 3, and the sheet size was controlled. They found that both sheet size and Cu/Al ratio influence the CO_2_RR selectivity. Sargent et al. prepared de-alloyed Cu-Al aggregates in micro scale on carbon papers, with the molar concentrations of Al on surfaces between 4.5% and 25%. They highlighted the importance of electrolyte-optimization strategy for multi-carbon production *via* CO_2_ electroreduction. Hence, in these two papers, they used different techniques to prepare Cu-Al materials with different properties, showing distinct performance for CO_2_RR. This outcome could be related to the Cu/Al ratio, morphology or test conditions. In our work, the Cu-Al materials were prepared with the same method through a microwave-assisted solvothermal route. The Cu_2_O-Al-3, Cu_2_O-Al-5 and Cu_2_O-Al-9 samples show a similar surface Cu/Al ratio but different particle sizes. The Cu_2_O-Al-3 performs better than the other two, and this could be attributed to its smaller size. Cu_2_O, Cu_2_O-Al-1 and Cu_2_O-Al-2 show similar cubic morphology but with very different surface Cu/Al ratios. With increase in Al content, the CO_2_RR selectivity is enhanced. Hence, both the Al content and particle size influence the performance of the catalysts. The total geometric current density (*j*_total_) of various electrode at different potentials are shown in Supplementary Figure S3. All Cu_2_O-Al-x samples show similar *j*_total_ at each potential, which is much higher than the one obtained on Cu_2_O electrode at the same potential.

**FIGURE 6:**
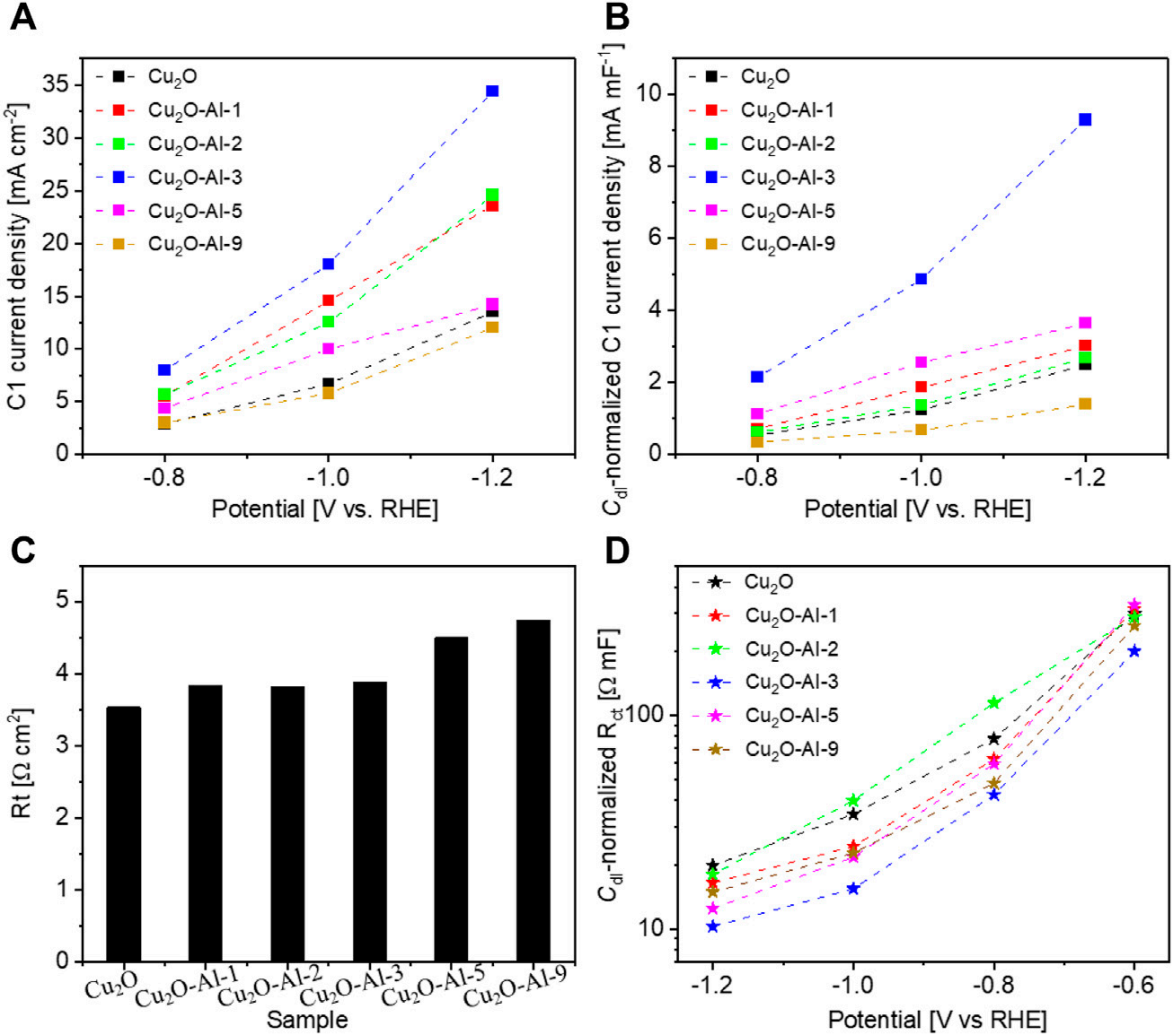
Comparison of various electrodes: **(A)** geometric current density devoted to C1 products; **(B)** *C*_dl_-normalized current density for C1 products; **(C)** Charge transport resistance and **(D)** *C*_dl_-normalized charge transfer resistance.

In order to understand better the catalytic performance of various catalysts, the geometric current density devoted to C1 products (CO and HCOOH, *j*_C1_) is compared in Figure 6A. Similar with the trend of *j*_total_, the *j*_C1_ is enhanced with adding a small amount of Al, peaking at the Cu_2_O-Al-3 electrode, and then decreases with further raising the Al percentage. To elucidate the intrinsic activity of the catalysts, the current should be normalized by the electrochemically active surface area (ECSA). The ECSA represents one of the most important properties of an electrode in the electrocatalysis. Besides cyclic voltammetry (CV), EIS is considered another powerful technique to determine the ECSA of an electrode (Reid et al., 2013). For this purpose, EIS has been performed on the Cu_2_O and Al-modified Cu_2_O electrodes. The impedance curves acquired on Cu_2_O-Al-3 electrode at different potentials are reported in Supplementary Figure S4 as an example. By fitting the EIS data through the equivalent circuit shown in the inset of Supplementary Figure S4, the electrical parameters, including double-layer capacitance (*C*_dl_), of various electrodes are obtained. All the parameters but the charge transfer resistance are found to be independent on the applied potential, in agreement with previous studies (Zeng et al., 2021c; Lourenço et al., 2021; Zeng et al., 2022b), and are reported in Supplementary Table S1. Since the ECSA is considered to be proportionally associated to the double-layer capacitance *C*_dl_, the intrinsic activity of various materials can be compared by investigating the *C*_dl_-normalized current densities. As shown in Figure 6B, the Cu_2_O-Al-3 catalysts exhibits much higher electrocatalytic activity toward the CO_2_RR to C1 products with respect to the counterparts. This outcome could be attributed to the good electrical conductivity of the Cu_2_O-Al-3 catalyst, which is inversely proportional to the transport resistance shown in Figure 6C, and to the low charge transfer resistance at the electrode/electrolyte interface exhibited in Figure 6D.

C1 products including CO and HCOOH are the main CO_2_RR products on the Cu_2_O-Al catalysts in KHCO_3_ electrolyte. The mechanism study of CO_2_RR on metal-based materials is widely studied, in combination of *in-situ* spectroscopic analyses and DFT calculations (Rosen et al., 2015; Genovese et al., 2017; Qin et al., 2018; Zhao et al., 2019). It is widely suggested that the CO_2_RR to CO process includes four elementary reaction steps: 1) one electron transfers to CO_2_ to form CO_2_^*−^; 2) one proton transfers to CO_2_^*−^ to obtain COOH^*^ intermediate; 3) an electron and a proton transfer to COOH^*^ to form CO^*^; 4) CO^*^ desorbs to produce CO. Another possible pathway is supposed to include three main steps: 1) an electron coupled with a proton transfers to CO_2_ to form COOH^*^ intermediate; 2) another electron coupled with a proton transfers to COOH^*^ to form CO^*^; 3) CO^*^ desorbs to produce CO. The formation of formic acid generally goes through the following pathway: 1) CO_2_^*−^ radical anion is firstly formed *via* a one-electron transfer and bonded to the electrode surface through O atom, 2) protonation of CO_2_^*−^ on the carbon atom leads to the formation of a HCOO^*^ intermediate and 3) a second electron transfer and protonation step results in the HCOOH product.

**FIGURE 7:**
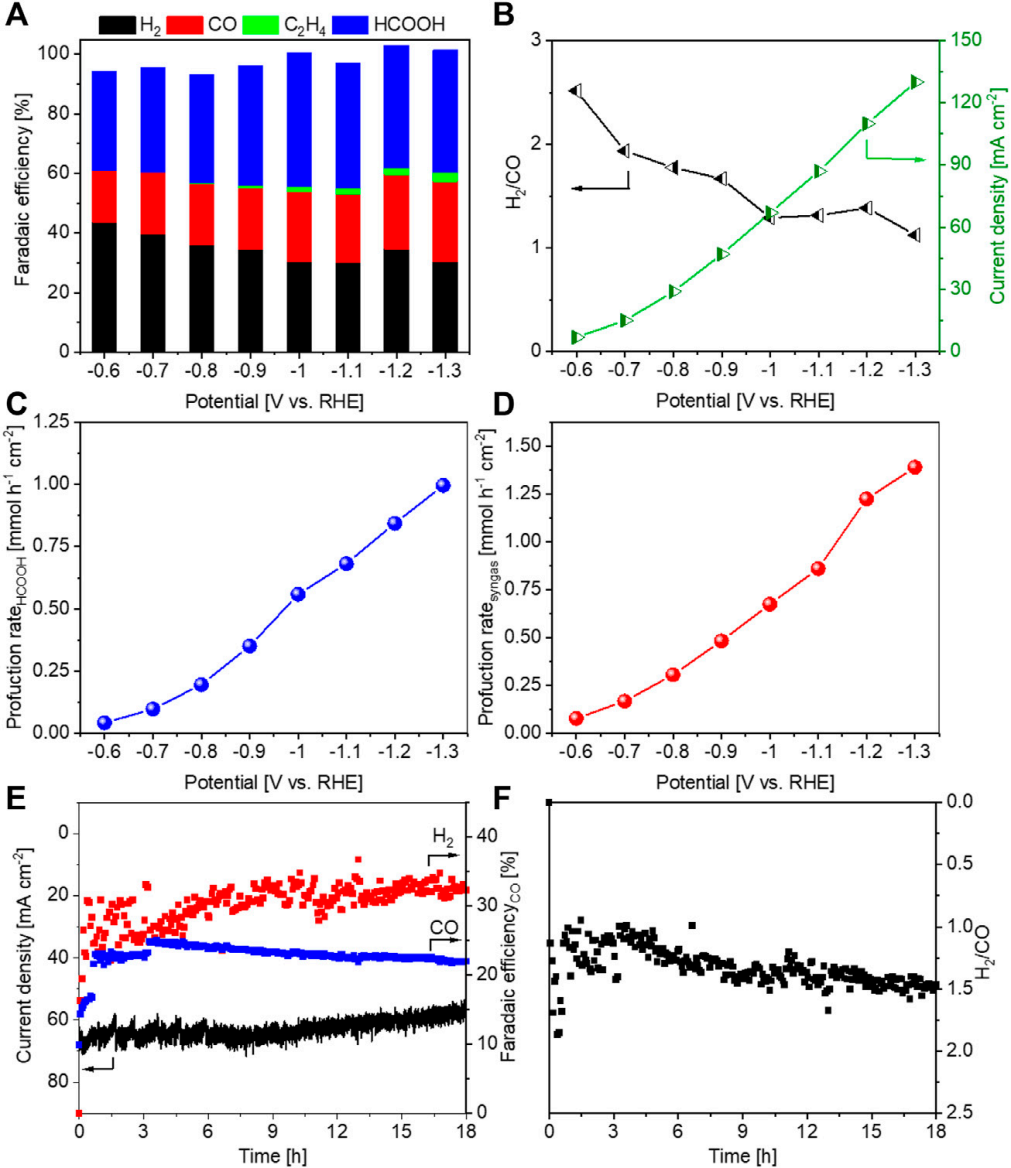
Sample Cu_2_O-Al-3 tested in a flow cell with 2.0 M KHCO_3_ electrolyte. **(A)** FE values for main products, **(B)** H_2_/CO ratios of syngas and geometric current densities, **(C)** production rate of HCOOH, **(D)** production rate of syngas, **(E)** a stability test and **(F)** H_2_/CO ratios of syngas during the stability test.

The best-performing Cu_2_O-Al-3 sample was further studied with 2.0 M KHCO_3_ electrolyte. As shown in Figure 7A, the FE_HCOOH_ ranges from 33% to 44%, and FE_CO_ varies between 17% and 26% at all the investigated potentials. A syngas with H_2_/CO ratio between 1 and 2 is formed at all potentials more negative than −0.6 V, and the current density is boosted in 2.0 M KHCO_3_ electrolyte with respect to that in the 0.5 M one, due to the higher conductivity of the former (Figure 7B). In addition, a more concentrated KHCO_3_ electrolyte leads to higher CO_2_ availability near the active sites (Zeng et al., 2022a), thus resulting in higher reaction rate and larger current density. The production rates for HCOOH and syngas achieve 0.56 and 0.67 mmol h^−1^ cm^−2^, respectively, at −1.0 V, and they increase up to 1.0 and 1.4 mmol h^−1^ cm^−2^, respectively, at −1.3 V, as reported in Figures 7C,D.

A long-term test has been perform on the Cu_2_O-Al-3 sample at −1.0 V with 2.0 M KHCO_3_ electrolyte. As can be seen from Figure 7E, FE_CO_ decreases, and FE_H2_ increases as a function of time. The current density decreases gradually during the test. Correspondingly, the H_2_/CO ratio rises but it remains between 1 and 1.5 during all the test (Figure 7F). Hence, the sample shows average stability at −1.0 V during an 18-h test. The degradation for the performance could be due to the restructuring of the material (Nitopi et al., 2019) or the formation of salts on the backside of the electrode (Sedighian Rasouli et al., 2020).

**FIGURE 8:**
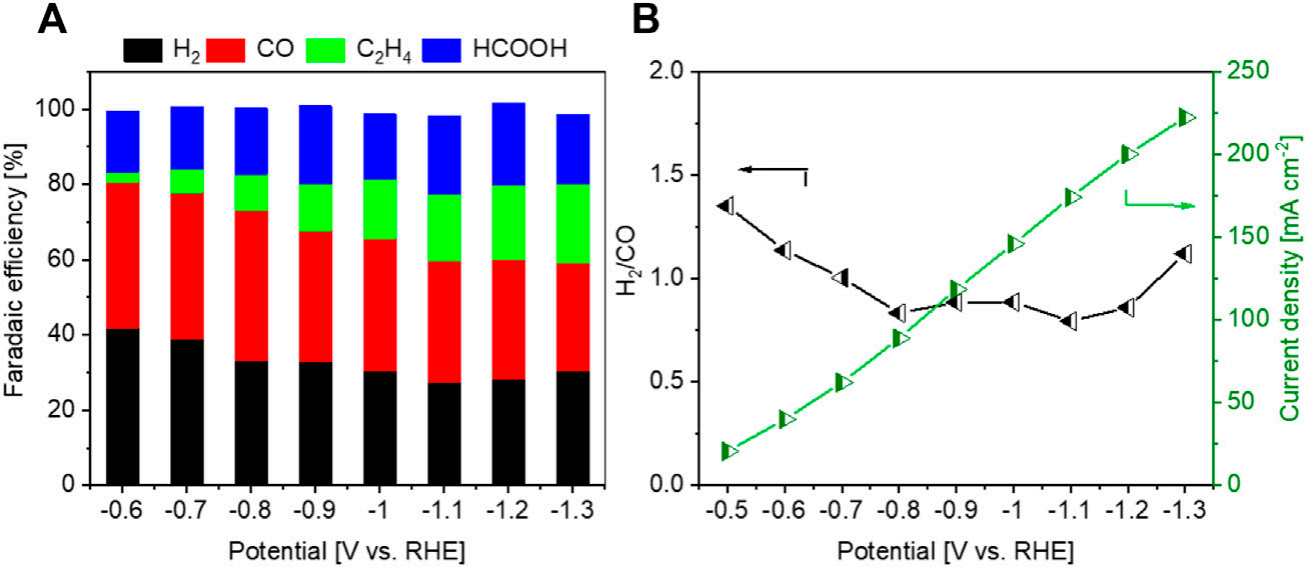
Sample Cu_2_O-Al-3 tested in a flow cell with 1.0 M KOH electrolyte. **(A)** FE values for main products, **(B)** H_2_/CO ratios of syngas and geometric current densities.

It is also observed that the selectivity for C_2_H_4_ production remains below 3.0% in 2.0 M KHCO_3_. Compared with the results reported by Honma et al. (Iwase et al., 2022) and by Sargent et al. (Zhong et al., 2020), it is likely that the employed electrolyte plays a vital role on the selectivity. To understand better this aspect, the Cu_2_O-Al-3 was further tested in a flow cell with 1.0 M KOH electrolyte. As shown in Figure 8, the C_2_H_4_ selectivity is significantly enhanced at all investigated potentials, ranging from 1.6% at −0.6 V to 21.0% at −1.3 V. The FE for HCOOH remarkably decreases, while those for CO and H_2_ as well as the H_2_/CO ratios are insignificantly altered. Regarding the electrode activity, the geometric current density is almost doubled in the KOH electrolyte with respect to that obtained in the 2.0 M KHCO_3_ one at each potential. These outcomes confirm the important roles of the employed electrolyte on the activity and selectivity of CO_2_RR. The KOH electrolyte has enhanced conductivity compared to KHCO_3_ one, due to the higher mobility of the ions, leading to higher reaction rates. Some studies also showed that surface hydroxyls offer effective sites to boost CO_2_ adsorption *via* hydrogen bond, enhancing the CO_2_RR activity and selectivity (Deng et al., 2019). The higher selectivity for C_2+_ products in KOH is attributed to the higher pH at the electrocatalytic interface, which promotes the dimerization of ^*^CO (Jouny et al., 2018). The herein obtained results, in agreement with those reported in literature, highlight the importance of the choice of electrolyte besides of catalysts for tuning the CO_2_RR toward targeted products. Moreover, the CO_2_RR performance of the Cu-Al catalyst in this work is in line with those reported in the literatures, as shown in Supplementary Table S2.

## Conclusion

In this work, Cu-Al bimetallic materials with various Cu/Al ratios were synthesized and proposed for catalyzing the CO_2_RR to HCOOH and syngas. The optimized Cu-Al catalyst achieves good selectivity and high activity for the targeted products. Most particularly, the good selectivity of HCOOH and the H_2_/CO ratio of syngas are maintained in a wide range of applied potentials. In 2.0 M KHCO_3_ electrolyte, the production rates for HCOOH and syngas achieve good values of 1.0 and 1.4 mmol h^−1^ cm^−2^ at −1.3 V, respectively. Both HCOOH and syngas are important C1-building blocks that are highly relevant for the chemical industry and have large market sizes. The herein proposed Cu-Al materials are prepared with a cost and time effective method, which is also environmentally friendly and energetically convenient, allowing their mass-scale production. Hence, they show good potential to be implemented in large-scale CO_2_ electrolysis technologies for mass production of C1 chemicals. Further studies of Cu-Al in KOH electrolyte show enhanced selectivity for C_2_H_4_, highlighting the vital role of electrolyte in the CO_2_RR besides catalysts.

## Data Availability

The original contributions presented in the study are included in the article/Supplementary Material, further inquiries can be directed to the corresponding author.
