# Supplementary material for: Electrochemical Reduction of CO2 With Good Efficiency on a Nanostructured Cu-Al Catalyst
Source: Front Chem. 2022 Jul 7;10:931767. doi: 10.3389/fchem.2022.931767 (PMC9300885; doi:10.3389/fchem.2022.931767)
Supplement: Supplementary file 1 [file DataSheet1.docx]

Electrochemical reduction of CO_2_ with good efficiency on a nanostructured Cu-Al catalyst

Juqin Zeng,^*a^ Micaela Castellino,^b^ Marco Fontana,^b^ Adriano Sacco,^a^ Nicolò B. D. Monti,^a,b^ Angelica Chiodoni, ^a^ Candido F. Pirri,^a,b^

^a^Center for Sustainable Future Technologies @POLITO, Istituto Italiano di Tecnologia, Via Livorno 60, 10144 Turin, Italy

^b^Department of Applied Science and Technology, Politecnico di Torino, C.so Duca degli Abruzzi 24, 10129 Turin, Italy

E-mail address of each author: [juqin.zeng@iit.it](mailto:juqin.zeng@iit.it), [micaela.castellino@polito.it](mailto:micaela.castellino@polito.it), [marco.fontana@polito.it](mailto:marco.fontana@polito.it), [adriano.sacco@iit.it](mailto:adriano.sacco@iit.it), [nicolo.monti@iit.it](mailto:nicolo.monti@iit.it), [angelica.chiodoni@iit.it](mailto:angelica.chiodoni@iit.it), [fabrizio.pirri@polito.it](mailto:fabrizio.pirri@polito.it)

^*^Corresponding author: juqin.zeng@iit.it


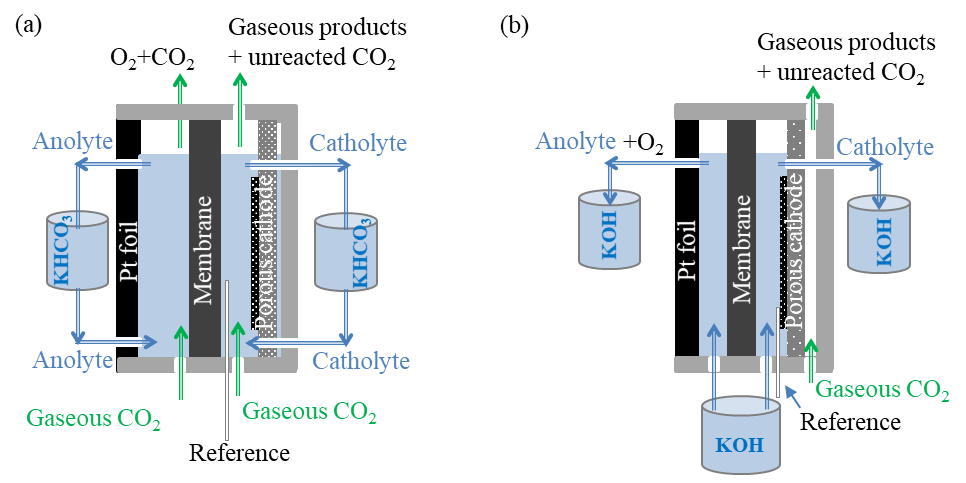


Scheme S1 Illustrations of the electrochemical cells: (a) three-electrode two-compartment configuration; (b) three-electrode three-compartment configuration


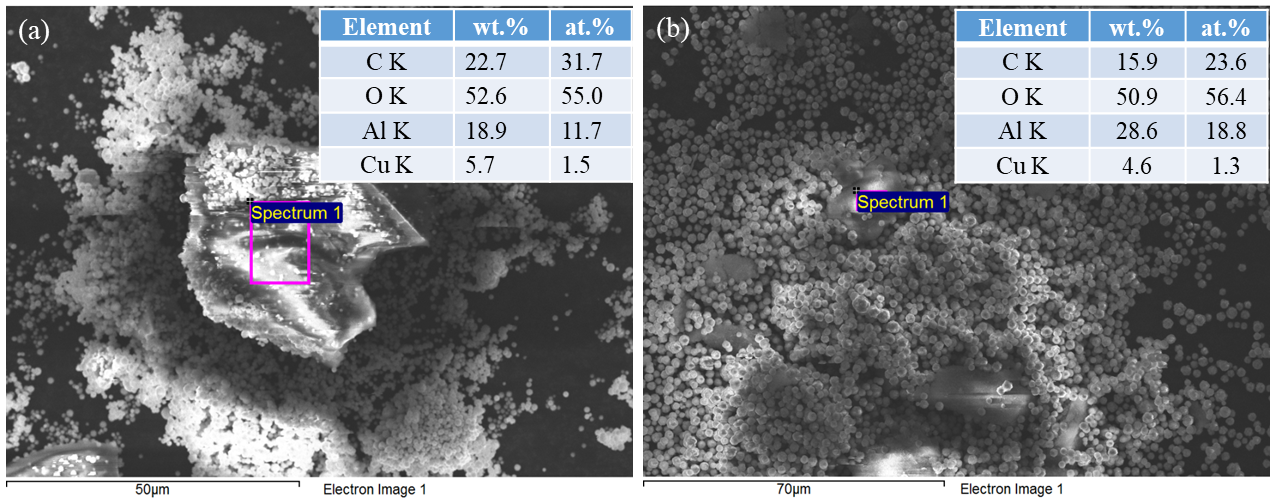


Figure S1 FESEM images and EDX of Cu-Al samples. (a) Cu_2_O-Al-5 and (b) Cu_2_O-Al-9.


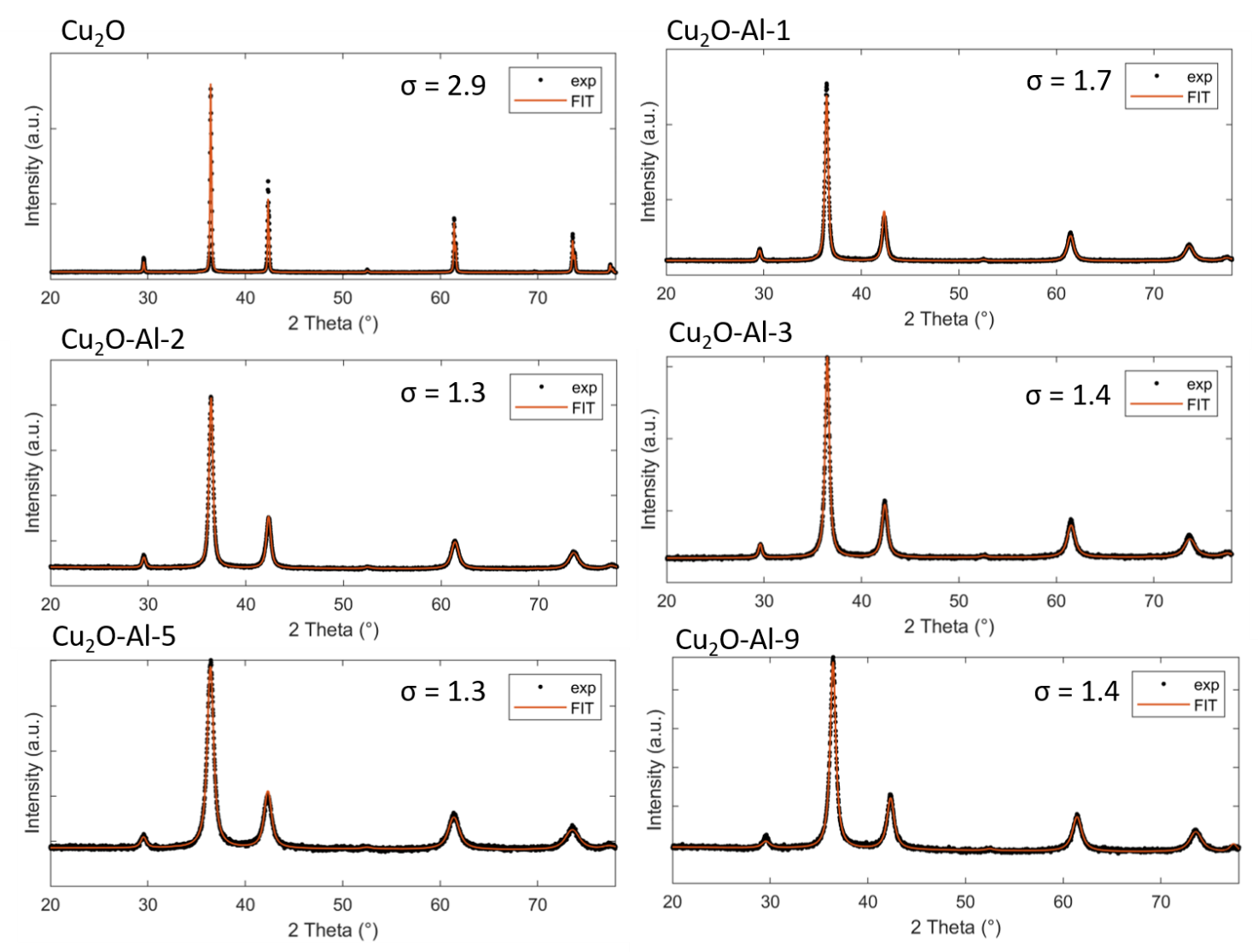


Figure S2 Rietveld refinement plots (“FIT” red line) vs experimental data (“exp”) for all the samples, alongside goodness of fit estimation (“σ” value).


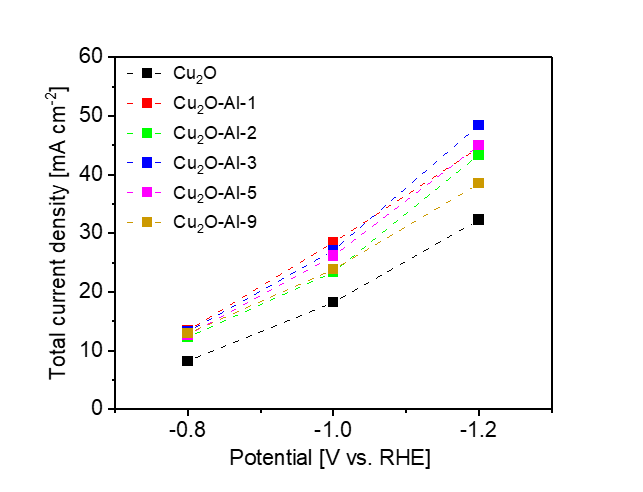


Figure S3 Total geometric current densities on various electrodes at different potentials.





Figure S4 Nyquist plot of the impedance of Cu_2_O-Al-3 electrode at various potentials. The points are experimental data, the continuous lines are calculated from simulation using the equivalent circuit reported in the inset (*R*_s_ – series resistance, *R*_t_ – transport resistance, *R*_ct_ charge transfer resistance, *C*_t_ – transport capacitance, *C*_dl_ – double layer capacitance). The other inset shows the high magnification of the 0 – 15 Ω cm^2^ region.

Table S1 Electrical parameters from EIS fitting.

| **Material** | ***R*_s_** | ***R*_t_** | ***C*_t_** | ***C*_dl_** |
| --- | --- | --- | --- | --- |
|  | **(Ω cm^2^)** | **(Ω cm^2^)** | **(µF cm^-2^)** | **(mF cm^-2^)** |
| **Cu_2_O** | 0.6 | 3.5 | 23.4 | 5.4 |
| **Cu_2_O-Al-1** | 0.3 | 3.8 | 21.3 | 7.8 |
| **Cu_2_O-Al-2** | 0.2 | 3.8 | 28.6 | 9.1 |
| **Cu_2_O-Al-3** | 0.4 | 3.9 | 20.9 | 3.7 |
| **Cu_2_O-Al-5** | 0.6 | 4.5 | 23.8 | 3.9 |
| **Cu_2_O-Al-9** | 0.3 | 4.7 | 23.4 | 8.6 |

Table S2 Comparison of the CO_2_RR performance of different Cu- and Al-based bimetallic electrocatalysts in aqueous electrolytes.

| Catalyst | Reaction product | Electrolyte | Potential  (V *vs* RHE) | Partial current density  (mA cm^-2^) | FE  (%) | Reference |
| --- | --- | --- | --- | --- | --- | --- |
| ZnCu | CO | 0.5 M KHCO_3_ | -0.95 | 4.5 | 90 | [1] |
| CuSn | CO | 0.1 M KHCO_3_ | -0.45 | 4.5 | 96 | [2] |
| CuIn | CO | 0.1 M KHCO_3_ | -0.60 | 1.5 | 90 | [3] |
| CuAu | CO | 0.1 M KHCO_3_ | -0.77 | 1.4 | 80 | [4] |
| PdCu | CO | 0.1 M KHCO_3_ | -0.89 | 6.9 | 86 | [5] |
| CuAg | CH_4_ | 0.1 M KHCO_3_ | -1.20 | 4.5 | 65 | [6] |
| CuBi | CO | 0.1 M KHCO_3_ | -0.90 | 0.3 | 24 | [7] |
| CuBi | HCOOH | 0.1 M KHCO_3_ | -0.90 | 2.0 | 90 | [7] |
| CuCd | CO | 0.1 M KHCO_3_ | -1.00 | 6.7 | 84 | [8] |
| CuFe | CO | 0.5 M KHCO_3_ | -0.40 | 0.5 | 95 | [9] |
| SbCu | CO | 2.0 M KHCO_3_ | -0.80 | 37.3 | 96 | [10] |
| CuAl | CO | 1.0 M KHCO_3_ | 50 mA (*) | - | 42 | [11] |
| CuAl | HCOOH | 1.0 M KHCO_3_ | 50 mA (*) | - | 22 | [11] |
| CuAl | C_2_H_4_ | 1.0 M KOH | -1.50 | 320.0 | 80 | [12] |
| CuAl | C_2_H_4_ | 1.0 M KHCO_3_ | 300 mA cm^-2^ (*) | - | 45 | [13] |
| CuAl | CO | 2.0 M KHCO_3_ | -1.30 | 32.5 | 25 | This work |
| CuAl | HCOOH | 2.0 M KHCO_3_ | -1.30 | 61.1 | 47 | This work |
| CuAl | C_2_H_4_ | 1.0 M KOH | -1.30 | 47.3 | 21 | This work |

(*) galvanostatic conditions

References:

[1] P. Moreno-García, N. Schlegel, A. Zanetti, A. C. López, M. de Jesús Gálvez-Vázquez, A. Dutta, M. Rahaman, P. Broekmann, ACS Appl. Mater. Interfaces 10 (2018) 31355-31365.

[2] C. J. Yoo, W. J. Dong, J. Y. Park, J. W. Lim, S. Kim, K. S. Choi, F. O. O. Ngome, S.-Y. Choi, J.-L. Lee, ACS Appl. Energy Mater. 3 (2020) 4466-4473.

[3] S. Rasul, D. H. Anjum, A. Jedidi, Y. Minenkov, L. Cavallo, K. Takanabe, Angewandte Chemie 54(7) (2015) 2146-2150.

[4] D. Kim, C. Xie, N. Becknell, Y. Yu, M. Karamad, K. Chen, E. J. Crumlin, J. K. Nørskov, P. Yang, J. Am. Chem. Soc. 139(24) (2017) 8329-8336.

[5] Z. Yin, D. Gao, S. Yao, B. Zhao, F. Cai, L. Lin, P. Tang, P. Zhai, G. Wang, D. Ma, X. Bao, Nano Energy 27 (2016) 35-43.

[6] W. J. Dong, C. J. Yoo, J. W. Lim, J. Y. Park, K. Kim, S. Kim, D. Lee, J.-L. Lee, Nano Energy 78 (2020) 105168.

[7] Z. B. Hoffman, T. S. Gray, Y. Xu, Q. Lin, T. B. Gunnoe, G. Zangari, ChemSusChem 12(1) (2019) 231-239.

[8] C. Wang, M. Cao, X. Jiang, M. Wang, Y. Shen, Electrochim. Acta 271 (20189 544-550.

[9] F. Wang, H. Xie, T. Liu, Y. Wu, B. Chen, Appl. Energy 269 (20209 115029.

[10] J. Zeng, M. Re Fiorentin, M. Fontana, M. Castellino, F. Risplendi, A. Sacco, G. Cicero, M.Amin Farkhondehfal, F. Drago, C. F. Pirri, Appl. Catal. B Environ. 306 (2022) 121089.

[11] K. Iwase, T. Hirano, I. Honma, ChemSusChem 15(2) (2022) e202102340.

[12] M. Zhong, K. Tran, Y. Min, C. Wang, Z. Wang, C.-T. Dinh, P. De Luna, Z. Yu, A. S. Rasouli, P. Brodersen, S. Sun, O.Voznyy, C.-S. Tan, M. Askerka, F. Che, M. Liu, A. Seifitokaldani, Y. Pang, S. C. Lo, A, Ip, Z. Ulissi, E. H. Sargent, Nature 581 (2020) 178-183.

[13] R. S. Kanase, K. B. Lee, M. Arunachalam, R. P. Sivasankaran, J. Oh, S. H. Kang, Appl. surf. sci. 584 (2022) 152518.
